# Nanocapsular Dispersion of Cinnamaldehyde for Enhanced Inhibitory Activity against Aflatoxin Production by *Aspergillus flavus*

**DOI:** 10.3390/molecules20046022

**Published:** 2015-04-07

**Authors:** Hongbo Li, Qingshan Shen, Wei Zhou, Haizhen Mo, Daodong Pan, Liangbin Hu

**Affiliations:** 1 School of Marine Science, Ningbo University, Ningbo 315211, China; E-Mail: hongbo715@163.com; 2 Department of Food Science, Henan Institute of Science and Technology, Xinxiang 453003, China; E-Mails: sqs973@hotmail.com (Q.S.); wzh_hist@hotmail.com (W.Z.); mohz@163.com (H.M.)

**Keywords:** cinnamaldehyde, nano-dispersion, aflatoxin, inhibition, peanut butter

## Abstract

Cinnamaldehyde (CA) is marginally soluble in water, making it challenging to evenly disperse it in foods, and resulting in lowered anti-*A. flavus* efficacy. In the present study, nano-dispersed CA (nano-CA) was prepared to increase its aqueous solubility. Free and nano-dispersed CA were compared in terms of their inhibitory activity against fungal growth and aflatoxin production of *A. flavus* both in Sabouraud Dextrose (SD) culture and in peanut butter. Our results indicated that free CA inhibited the mycelia growth and aflatoxin production of *A. flavus* with a minimal inhibitory concentration (MIC) value of 1.0 mM, but promoted the aflatoxin production at some concentrations lower than the MIC. Nano-CA had a lower MIC value of 0.8 mM against *A. flavus*, and also showed improved activity against aflatoxin production without the promotion at lower dose. The solidity of peanut butter had an adverse impact on the antifungal activity of free CA, whereas nano-dispersed CA showed more than 2-fold improved activity against the growth of *A. flavus*. Free CA still promoted AFB_1_ production at the concentration of 0.25 mM, whereas nano-CA showed more efficient inhibition of AFB1 production in the butter.

## 1. Introduction

*A. flavus* is a well-known diffused fungus that is able to produce aflatoxin (AF), thus contaminating food commodities and feeds [1]. Exposure to low doses of AF can increase the risk of liver cancer in people chronically infected with hepatitis B virus (HBV), while high doses are acutely toxic and lethal [2]. The aflatoxin problem resulting from mouldy foods is longstanding, and has been recognized as ubiquitous, especially in developing countries [3]. 

Numerous diverse compounds and extracts showing activity inhibitory to aflatoxin biosynthesis have been reported, including alkaloids, antibiotics, bioflavonoids, Ca^2+^ blockers, coumarins, flavonoids, hydroxamic acids, phenolics, oxylipins, terpenoids, volatiles, glucans and DNA methylation inhibitors [4,5,6]. However, given sufficient time in culture, most inhibitors are eventually overcome [4], so agents active against both the fungal growth and AF production have more potential in the control of AF contamination.

Cinnamaldehyde (CA) is one of the most effective compounds existing in cinnamon [7] and has applied widely in food stuff preservation as a safe antimicrobial ingredient [8]. CA has great antifungal activity against wood-rot fungi, and it also inhibits the growth of *P. capsici* by stimulating a transient Ca^2+^ efflux [9,10]. Morozumi found that *o*-methoxycinnamaldehyde could completely inhibit the growth of *A. parasiticus* and *A. flavus* at 100 μg/mL, and inhibited the production of aflatoxin B_1_ by over 90% at 6.25 μg/mL [11]. CA can enter fungal cells by damaging the cell membrane, so as to change its biomacromolecules’ spatial structures, destroying its ordered metabolism and inhibiting the mycotoxin growth [12]. However, due to its hydrophobicity, CA is sparingly soluble in water and it is difficult to uniformly disperse in food matrices. This leads to a relatively limited application of CA in food preservation. To solve this problem CA has been delivered via carrier-solutions, polymer derivatives, or encapsulated in solid particles. Rieger demonstrated that chitosan/CA/PEO nanofiber mats can serve as CA delivery vehicles that can potentially eradicate *Pseudomonas* infections [13,14]. Nanocapsular dispersion invoves dissolving CA in a solvent mixture to change its polarity and disperse it as emulsion droplets. Because of Brownian motion, these tiny nanoparticles stay kinetically stabilized and are able to mix evenly with food ingredients [7]. In the present study, we prepared nano-dispersions of CA (nano-CA), and evaluated the improved inhibitory activity of nano-CA against the fungal growth and aflatoxin production of *A. flavus*.

## 2. Results and Discussion

### 2.1. Properties of the Nano-Dispersion 

**Figure 1:**
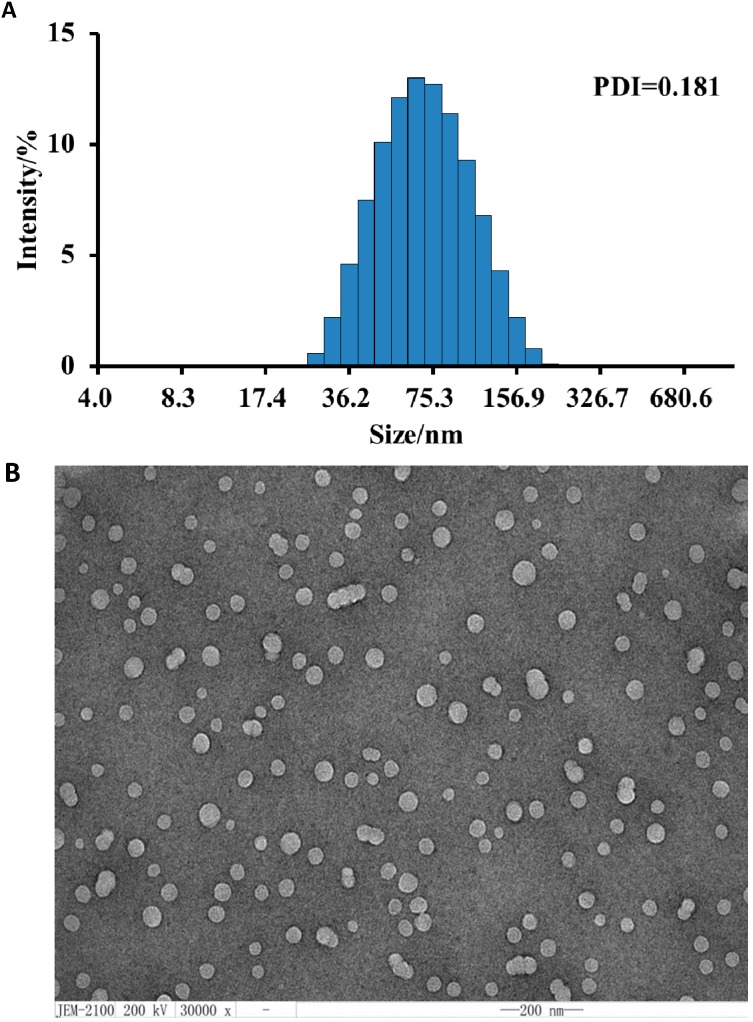
Characterization of particles in nano-dispersed CA. (**A**) Particle size distribution measured by laser granulometry (Zetasizer Nano-ZS90, Malvern, WR14 1XZ, UK); (**B**) Imaging of nano-dispersed CA under TEM, showing a high abundance of nanoparticles with a similar diameter.

The nano-dispersion of CA was prepared at a particular concentration of oil, surfactant and water corresponding to their ratios described in the Experimental Section and formed a stable system with no phase separation, creaming or cracking. This system could remain stable at temperatures ranging from 4 °C to 45 °C. The sizes of particles in this dispersion were determined using a Zetasizer. The results indicated that the mean particle size in this system was 66.1 nm, and PDI was 0.181 (Figure 1A). The particle size also could be assessed by TEM. CA-capsuling particles appeared dark and their surroundings were bright (Figure 1B). Their sizes agreed with the Zetasizer results. Taken together, these results confirmed the nano-dispersion of CA was well prepared. The nano-dispersion contained 30 g/L CA, which was well above the high aqueous insolubility limit of CA. It was notable that the nano-CA released a far weaker smell, which suggested that the CA molecules were tightly captured in the nano-particles. 

### 2.2. Improved Inhibitory Activity against A. flavus

**Figure 2:**
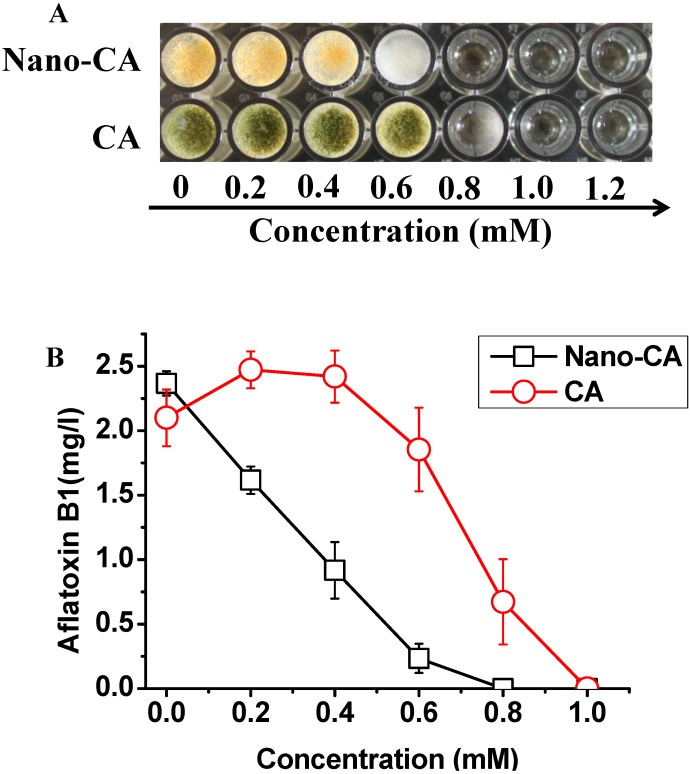
Inhibitory effects of free and nano-dispersed CA on the growth (**A**) and AFB1 production (**B**) of *A. flavus* in culture. The control wells of CA treatment and nano-CA treatment contained DMSO and nano-emultion mixture solution without CA, respectively. Three independent cultivations were performed, and bars denote standard deviations.

Due to its activity against bacteria, yeast, and filamentous molds, CA has been developed as a food antimicrobial agent [15,16]. Our results confirmed that CA could efficiently inhibit the growth of *A. flavus* with a visible MIC value of 1.0 mM (Figure 2A). However, CA could promote the aflatoxin production at some concentrations lower than the MIC (Figure 2B), which was detrimental for its potential application as an anti-aflatoxin agent in food. Several studies have showed the antimicrobial activity of CA was associated with its induction of oxidative stress *in vivo* [17,18]. It has also been confirmed that oxidative stress leads to more aflatoxin production [19,20], so it can be proposed that oxidative stress could be triggered by CA at low doses without showing any inhibitory effect on mycelia growth, which in turn promoted aflatoxin production. 

**Figure 3:**
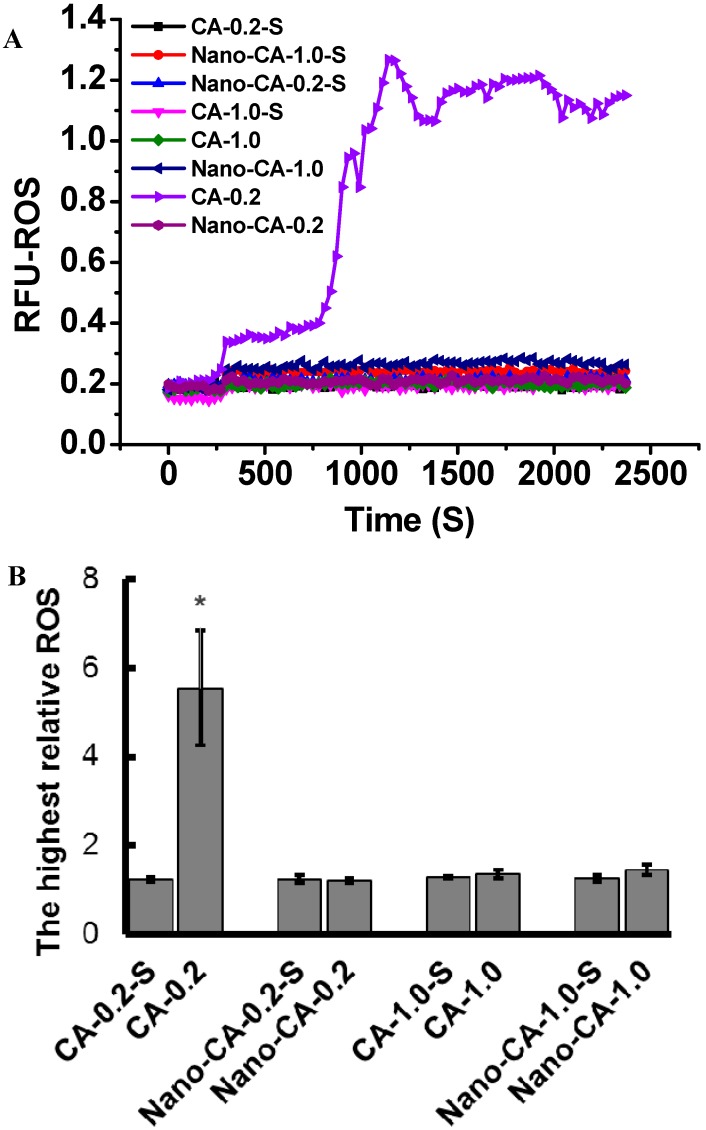
Effects of CA and nano-CA on the ROS production in the mycelia of *A. flavus*. (**A**) time course of relative fluorescence intensity indicating ROS; All tested agents were added post incubation at 30 °C for 270 s. A representative trace of three repeats of each experiment was shown. (**B**) Highest relative ROS post-addition of different agents (the highest relative fluorescence intensity of ROS/the original fluorescence intensity of ROS). Asterisk indicates that mean values of three replicates are significantly different from the other groups (*p* < 0.05). CA-0.2: 0.2 mM CA; CA-0.2-S: solvent for 0.2 mM CA; CA-1.0: 1.0 mM CA; CA-1.0-S: solvent for 1.0 mM CA; Nano-CA-0.2: nano-CA dispersion containing 0.2 mM CA; Nano-CA-0.2-S: nano-CA-0.2 without CA; Nano-CA-1.0: nano-CA dispersion containing 1.0 mM CA; Nano-CA-1.0-S: nano-CA-1.0 without CA.

Unexpectedly, reactive oxygen species (ROS) in *A. flavus* were not induced by 1.0 mM CA (more than the MIC), but they erupted increasing more than 4-fold in the presence of 0.2 mM CA (lower than the MIC) (Figure 3A,B). This not only suggested CA inhibits the growth of *A. flavus* via a mechanism independent of ROS, but was also in accordance with the promotion of aflatoxin production by CA at concentrations lower than the MIC. Nano-emulations of CA could avoid being held up in the membrane due to their hydrophobicity, which led to improved traffic to the cytoplasm. This could contribute to the approach of CA to its targets. As a consequence, nano-CA showed improved inhibitory effects on the growth of *A. flavus*, which was confirmed by our results in Figure 2A. 

The results showed the nano-dispersion of CA had a lower MIC value of 0.8 mM against A. flavus (Figure 2A). It should be noted that nano-dispersions of CA did not promote the aflatoxin production at a concentration lower than the MIC (Figure 2B), which should be associated with its non-participation in ROS induction (Figure 3A,B). Taken together, our results support the fact that nano-CA has the potential to be applied in the control of AFB_1_ contamination.

### 2.3. Evaluation on the Prevention of Aflatoxin Contamination in Peanut Butter

**Figure 4:**
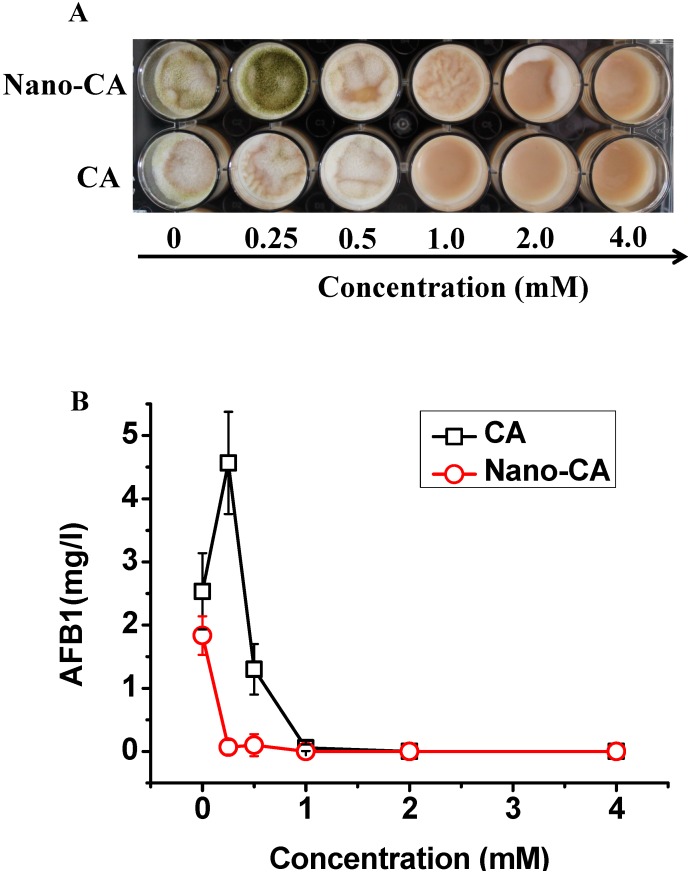
The evaluation of free and nano-dispersed CA in the prevention of *A. flavus* contamination in the peanut butter. (**A**) Visual fungal growth in the peanut butter; (**B**) AFB_1_ levels in the peanut butter. The control wells (0 mM) of CA treatment and nano-CA treatment contained DMSO and nano-emultion mixture solution without CA, respectively. Three independent cultivations were performed, and bars denote standard deviations.

Peanut butter, and paprika powder are subject to aflatoxin contamination [21,22]. Therefore, we chose peanut butter to evaluate the efficacy of nano-CA in the control of AFB_1_ contamination in foodstuffs. The solidity of the butter had an adverse impact on the antifungal activity of CA. It could be observed that no visible fungal growth occurred in the well until the CA concentration reached 4.0 mM (Figure 4A). 

Nano-CA showed more than 2-fold improved activity against the growth of *A. flavus* in the butter (Figure 4A), which was far higher than the 20% improvement in the culture (Figure 2A). This was partly due to the good dispersity of nano-CA in the butter. Although both CA and nano-CA showed more efficient in the inhibition to AFB_1_ production in the peanut butter, CA still showed promotion of AFB_1_ production at the concentration of 0.25 mM (Figure 4B). Thus it was preferable to apply nano-CA instead of free CA to control the aflatoxin contamination in peanut butter.

## 3. Experimental Section

### 3.1. Chemicals

Cinnamaldehyde (99%), and AFB_1_ standard (≥98%) were obtained from Aldrich (St. Louis, MO, USA). All the other chemicals were of reagent grade. 

### 3.2. Preparation and Properties of Nano-Dispersion

#### 3.2.1. Preparation of Nano-Dispersion

Firstly, Tween-80 (27.0 mL) was added to a beaker. At low magnetic stirring speed ethanol (2.16 mL), 1,2-propanediol (1.71 mL), extra virgin olive oil (BETIS, San Juan, Spain, 1.02 mL) and CA (2.81 mL) were slowly added into the beaker in order. Then the speed of magnetic stirrers was appropriately adjusted according to the viscosity. During the mixing process, distilled water (65.3 mL) was added dropwise to the mixture until the dispersion changed from turbid to clear. Finally, the speed of the magnetic stirrer was decreased gradually to release any bubbles. The prepared CA dispersion was collected and stored in a freezer at 4 °C [23]. 

#### 3.2.2. Thermodynamic Stability Studies

The prepared nano-CA was placed at 4 °C and 45 °C respectively for not less than 48 h. Those formulations, which were stable at both temperatures, were subjected to centrifugation tests at 3500× *g* for 30 min. 

#### 3.2.3. Particle Size Analysis

Five-fold dilution of the prepared nano-CA was used for the particle diameter measurements. The dilution was filtered through a 0.45 μm microporous membrane, and then its particle-size distribution was determined by laser granulometry (Zetasizer Nano-ZS90). 

#### 3.2.4. TEM Analysis

Transmission electron microscopy (TEM) (JEM-2100, JEOL, Tokyo, Japan) was utilized to characterize the particles in the prepared nano-dispersion. Five-fold dilution of the nano-CA was passed through a sterile membrane, and then 1 drop of dilution was directly deposited on the holey film grid for 10 min. Finally, 3% sodium phosphotungstate was added for negative staining. After drying for 10 min, the sample was observed under TEM. 

### 3.3. Culture of A. flavus 

*A. flavus* CGMCC3.2890 from the the China General Microbial Culture Collection Centre was cultured on Sabouraud Dextrose (SD) medium containing 4% (w/v) glucose, 1% peptone, and 2% agar for 3 d. Then spore suspension was prepared by slightly shaking the plate after the addition of 0.05% Triton X-100 and counted in the blood counting chamber under a microscope. 

### 3.4. A. flavus Cultured in SD Medium 

The SD liquid medium (without agar) was inoculated with the spores of *A. flavus* up to approximately 10^4^/mL, and then piped into the wells of 96-well plate (150 μL/well). Free CA and nano-CA were added into the wells at the indicated concentrations of 0.2, 0.4, 0.6, 0.8, 1.0 and 1.2 mM, respectively. The plate was incubated at 30 °C for 48 h, and then were assessed visually by comparison with the CA-free growth control of the plate. The CA-free control well was added with DMSO solvent and the nano-CA treatment control well was added with the nano-CA solution without CA. The MIC was defined as the minimum CA concentration that provided no visible growth in the wells [24]. In addition, the culture in each well was also sampled for the AFB_1_ assay. 

### 3.5. A. flavus Cultured in Peanut Butter 

Peanut butter of “Skippy” brand (Unilever Com., Wageningen, The Netherlands) was purchased from a local supermarket. A mixture of peanut butter and sterile water in a ratio (v/v) of 1:1 was inoculated with the spores of *A. flavus* (approximately 10^3^/mL), and then added into the wells of 24 wells plate (1 mL/well). Free CA and nano-CA were added into the wells at the indicated concentration of 0, 0.25, 0.5, 1.0, 2.0 and 4.0 mM, respectively. For the control wells, only the solvent DMSO or the nano-CA solution without CA was added. After incubation at 30 °C for 48 h, fungal growth was identified by observation of visual hyphal growth inside the well. Also, mixture in each well was sampled for the AFB_1_ assay. 

### 3.6. Aflatoxin Assay 

AFB_1_ in *A. flavus* culture or peanut butter mixture was extracted with chloroform in a ratio (v/v) of 1:3 (culture broth/chloroform). The extracts were dried with a nitrogen-blowing instrument (DN-12A, Dongkang Co., Ltd., Tianjin, China), followed by dissolution in methanol, and then filtered through a 0.22-μm microporous membrane for subsequent quantification. The AFB_1_ amount was determined using a high-performance liquid chromatography (HPLC) method detailed previously [5]. The final data of AFB_1_ content in samples was calculated against the AFB_1_ standard curve [25]. 

### 3.7. ROS Assay 

The intracellular reactive oxygen species (ROS) level was monitored using the Reactive Oxygen Species Assay Kit S033 (Beyotime, Shanghai, China) to assess the oxidative state *in vivo*. The SD liquid medium was inoculated with the spores of *A. flavus* up to approximately 10^4^/mL, and then incubated at 30 °C, 200 g for 5 h. After the addition of DCFH-DA, the developing mycelia-spores mixture was incubated at 30 °C, 200 g for 30 min in the dark. The organism of 1 mL mixture was collected by centrifugation at 5000× *g* for 7 min, and then washed twice with Hanksa balanced salt solution (HBSS). Finally, the organism was suspended in HBSS, and piped into a 96-well plate (100 μL/well). A given amount of free CA and nano-CA were added into the wells, and the fluorescence at 525 nm (excitation light at 488 nm) of each independent well was continuously monitored with a measure frequency of 30 s using a fluorescence plate reader (Thermo Fisher, Waltham, MA, USA). The continuous changes of ROS-dependent fluorescence for each trace were calculated use relative fluorescence units (RFU). A representative trace of three repeats of each experiment was shown. At the same time, the highest relative ROS post the addition of different agents were calculated by the ratio of the highest relative fluorescence intensity of ROS and the original fluorescence intensity of ROS.

### 3.8. Statistical Analysis 

All the presented data were the mean value ± standard errors of the means (SEM) of three determinations. One-way ANOVA was used to determine whether there were significant differences between the means in all of the experiments. The differences were considered to be significant if the *p* value was less than 0.05. 

## 4. Conclusions

Nano-dispersion of CA not only improved its solubility but increased its potential in the control of aflatoxin contamination of foodstuffs. This included improved efficacy against fungal mycelial growth and aflatoxin production and the removal of negative effects on the toxin control at low doses. In a complex food system, nano-dispersion of CA could probably be more efficient against aflatoxin contamination due to its good dispersity.
